# GLI1: A Therapeutic Target for Cancer

**DOI:** 10.3389/fonc.2021.673154

**Published:** 2021-05-25

**Authors:** Justin T. Avery, Ruowen Zhang, Rebecca J. Boohaker

**Affiliations:** ^1^ Oncology Department, Drug Discovery Division, Southern Research, Birmingham, AL, United States; ^2^ Department of Medicine, Stony Brook University, Stony Brook, NY, United States

**Keywords:** hedgehog, GLI1, therapeutic resistance, DNA damage repair, cancer

## Abstract

GLI1 is a transcriptional effector at the terminal end of the Hedgehog signaling (Hh) pathway and is tightly regulated during embryonic development and tissue patterning/differentiation. GLI1 has low-level expression in differentiated tissues, however, in certain cancers, aberrant activation of GLI1 has been linked to the promotion of numerous hallmarks of cancer, such as proliferation, survival, angiogenesis, metastasis, metabolic rewiring, and chemotherapeutic resistance. All of these are driven, in part, by GLI1’s role in regulating cell cycle, DNA replication and DNA damage repair processes. The consequences of GLI1 oncogenic activity, specifically the activity surrounding DNA damage repair proteins, such as NBS1, and cell cycle proteins, such as CDK1, can be linked to tumorigenesis and chemoresistance. Therefore, understanding the underlying mechanisms driving GLI1 dysregulation can provide prognostic and diagnostic biomarkers to identify a patient population that would derive therapeutic benefit from either direct inhibition of GLI1 or targeted therapy towards proteins downstream of GLI1 regulation.

## GLI and the Hedgehog Pathway

GLI1 is an effector transcriptional factor distal to both the canonical and non-canonical Hedgehog (Hh) signaling pathways. The Hh family of proteins contains three subfamilies: sonic hedgehog (SHh), desert hedgehog (DHh) and Indian hedgehog (IHh) (1). IHh and DHh are reported to be involved in normal tissue development, such as bone formation (2). SHh, first discovered in Drosophila, has been found to be highly conserved across many different vertebrate species including human, mouse, rat, frog, fish, and chicken, and is the most studied member of the hedgehog family (3). SHh plays a critical role in the embryonic development that is necessary for certain cell differentiation and maintenance of tissue polarity (4). Due to its conserved nature, and apparent critical functionality across organisms, SHh and the downstream pathway members have evolved to serve vastly diverse roles in both embryonic and non-embryonic cellular homeostasis. Herein, we focus specifically on our current understanding of SHh-GLI pathway and its clinical significance in human development and the consequences of its dysregulation in disease progression (5–8).

SHh-mediated transduction is initiated *via* extracellular SHh ligand binding to the 12-span transmembrane receptor, PATCHED-1 (PTCH-1) or the redundant receptor PTCH-2, in target cells (9, 10). In the absence of SHh, PTCH-1 and, redundantly, PTCH-2, catalytically inhibit downstream signaling activity with seven-transmembrane G-protein-coupled receptor, Smoothened (SMO) (11–13). Upon SHh binding to PTCH, the inhibitory interaction is terminated through internalization of PTCH, releasing SMO and allowing for phosphorylation to transduce signal into the cytoplasm (14). The resultant signal leads to the component dissociation of a large protein complex comprising of Sufu and GLIs in the cytoplasm, releasing the GLI transcription factors. Finally, the released GLI transcription factors translocate into the nucleus to execute transcriptional activation of specific target genes (15). Aberrant activation of the hedgehog pathway has been shown to promote oncogenic activities, such as metastasis, DNA damage repair, stem-ness, and chemotherapeutic resistance, in a variety of types of cancer (16–26).

There are two models for the over-activation of the Hh pathway in cancer: (a) ligand-dependent model: tumors are able to over-activate SHh-GLI pathway *via* autocrine signaling to produce high level of SHh ligands (18, 27–31). This can be observed in several epithelial originating tumors such as small cell lung cancer (SCLC), pancreatic, colon, and prostate cancer, and glioblastomas, and medulloblastomas. (b) Ligand-independent model: Clinical observations have found mutations of PTCH-1 and PTCH-2 in basal cell carcinomas and in medulloblastomas, resulting in dysregulated GLI signaling due to ineffective sequestration of SMO signaling, regardless of SHh ligand levels. Mutant PTCH often results in SMO constitutive activation, subsequently promoting cell transformation and tumorigenesis (32). Inactivation of PTCH-1 due to gene mutation has also been reported in trichoepitheliomas (33), esophageal squamous cell carcinomas (34), and transitional cell carcinomas of the bladder (35). In both models, the commonality is a failure to stifle SMO signal transduction.

Regardless of how SHh-GLI pathway is activated, all biological function of these upstream proteins such as SHh, PTCH and SMO depends on the transcriptional effectors at the distal end of the pathway: the GLI proteins. There are three GLI transcriptional proteins in this family, two which act as transcriptional activators (GLI1 and GLI2) and one transcriptional repressor (GLI3) (36). GLI1 was initially found to transcriptionally regulate specific target genes involved in mammalian development, such as patterning in the central nervous system, proliferation, differentiation, and survival (37). However, increased expression in terminally differentiated cells is a known oncogenic biomarker for numbers cancer subtypes (38–40), making it an ideal drug discovery target.

## GLI1 as a Transcription Factor

GLI1 (1106 amino acids; MW 117.9kDa) was originally identified as an amplified gene product in a malignant glioma (41) and was the first member described in the human GLI gene family. GLI1-DNA binding is mediated by five highly conserved tandem C2-H2 zinc finger (ZF) domains and a consensus histidine-cysteine linker sequence between zinc fingers (42). While ZF1-3 interacts with the phosphate backbone and contributes to binding stability and recruitment of co-regulatory factors, ZF4-5 regulates transcription, recognizing the consensus sequence 5'-GACCACCCA-3' in the promoter region of target genes. The two cytosine-pairs flanking the central adenine within the consensus site are critical for GLI binding, whereas the other positions can tolerate a certain degree of flexibility (43). In addition to the transcriptional ZF domain, the GLI proteins contain both nuclear export sequence (NES) and a nuclear localization signal (NLS), which facilitate the nucleo-cytoplasmic shuttling of GLI (44). GLI1 also contains a single SUFU-interacting site located at the N-terminus (SIN) (45), which is responsible for SUFU-mediated cytoplasmic retention of GLI1. The positioning of the SIN is unique to GLI1; GLI2 and GLI3 also have a SUFU-interacting site though it is located in the C-terminus (SIC) (45, 46). The GLI1 C-terminal region possesses a transactivation domain (TAD) which remodels chromatin and interacts with histone acetyltransferase (HAT), histone deacetylase (HDAC); SWI-SNF5; SWI/SNF-like Brg/Brm-associated factor; and the TFIID TATA box-binding protein-associated factor, TAFII31 (26). Like the SIN domain, all GLI proteins also possess a TAD, but GLI2 and GLI3 have an additional N-terminal repressor domain, which is lacking on GLI1. Therefore, GLI1 performs as a strong transcriptional activator (47), whereas full-length GLI2 is generally a weak activator since the fully activated form requires significant truncation of its N-terminus and C-terminus (48–51), and GLI3 has been reported as a strong repressor in most settings (52).

Two additional isoforms of GLI1, N-terminal deletion variant (GLI1ΔN) and truncated GLI1 (tGLI1), have been identified. GLI1ΔN is generation is the result of a 128-amino acid deletion on its N-terminus (47). This deletion results in loss of the lone critical suppressive SUFU-binding domain on the GLI1 protein sequence, while preserving the ZNF domains, NLS and NES, and the transactivation domain. As would be expected, this isoform of GLI1 functions as a constitutively active protein, with activity comparable to full-length GLI1 (GLI1FL) but surprisingly does not show a preferential expression in cancer tissues (53, 54). tGLI1 originates from a splicing of exon 3 and part of exon 4 of the GLI1 gene, resulting in the deletion of 41 amino acids (55). All functional domains are retained in tGLI1, and this isoform is observed specifically in tumor expression. It has been shown to regulate an additional set of target genes involved in EMT, invasion and metastasis (56). All three GLI1 isoforms (GLI1FL, GLI1ΔN, and tGLI1) could be activated by SHh ligand stimulation, but whether they induce differently transcriptional targets has not yet been determined.

## Non-Canonical Activation of GLI1

**Figure 1:**
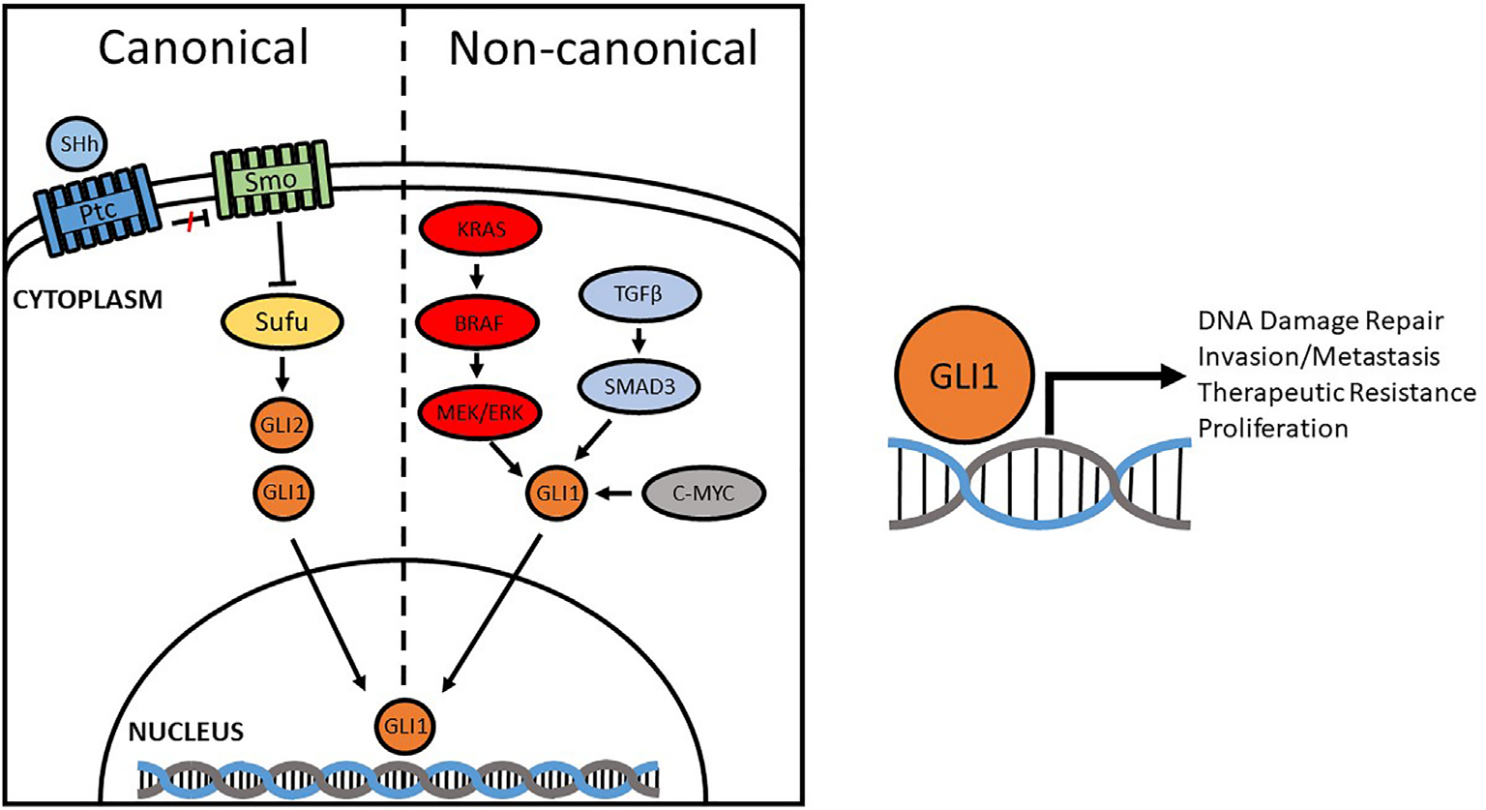
Canonical and Non-canonical activation of GLI1. Compounds originally designed to inhibit the Hedgehog pathway focused on canonical regulators, like SMO, but were found to be ineffective in some cancers due to non-canonical activation. Aberrant activation of GLI1 promotes DNA damage repair, invasion/metastasis, proliferation, and therapeutic resistance through transcriptional regulation of target genes.

Over-activation of Hh promotes the tumor microenvironment through pro-inflammatory mechanisms, angiogenesis, genome instability, mutation, resistance to cell death, energy imbalance, and is involved in invasion and metastasis (57, 58). Some studies, however, fail to observe a positive correlation between the Hh signaling pathway and the development/progression of cancer (59–61). For instance, Li discovered that SMO expression was not statistically correlated with CRC-specific or overall survival; the same results were reported by Stefanius, where no correlation between Hh and colorectal serrated adenocarcinomas was observed (62, 63). Our lab, like many others, observed a positive correlation between GLI1 expression and disease severity (64). We also demonstrated that both GLI1 and one of its transcriptional targets, NBS1, negatively correlate with CRC patient 5-year survival, driving chemotherapeutic resistance by overcoming FOLFOX induced DNA damage (standard of care treatment). The difference lies in the way GLI1 is activated—whether it be through canonical activation (PTCH/SMO) or non-canonical (RAS/RAF, etc) (**Figure 1**). Elevated levels of GLI1 in cancer are often driven by non-canonical pathways. As such, this explains why Vismodegib, the first SMO inhibitor to be approved by the FDA for the treatment of BCC (65), failed to demonstrate the effectiveness in clinical trials for the treatment of metastatic colorectal cancer where GLI expression is driven non-canonically (66). Therefore, it is important to determine how GLI1 is upregulated and its function in the initiation, progression, invasion and metastasis in order to develop a therapeutic target for new treatment schemes based on the inhibition, at different levels, of the Hh pathway (67–69).

### RAS-RAF-MEK-ERK Pathway

The RAS-RAF-MEK-ERK pathway is the most common non-canonical mechanism involved both in transcriptional activation of GLI genes and in post-translational modifications of GLI-transcribed proteins. In colorectal cancer, constitutively activated mutant KRAS or altered stimulation of pathway components (mainly RAS, RAF, MEK) results in the hyperactivation of the mitogen-activated protein kinase (MAPK) extracellular signal-regulated kinase 1 and 2 (ERK1/2) and positively modulates tumor proliferation by increasing GLI1 transcriptional activity and expression of Hh target genes (54, 70, 71). This non-canonical activation pathway was confirmed in a study where EGF-induced stimulation of GLI is unaffected by SMO inhibition but was blocked by MEK1 inhibition (72). Similarly, the RAS-RAF pathway induces GLI1 and GLI2 transcriptional activity and increases mRNA and protein levels in a non-canonical manner in colon cancer cells (73). Pharmacological and genetic inhibition of GLI function is more effective in reducing tumor proliferation and inducing apoptosis than the inhibition of the canonical pathway at SMO level, suggesting that GLI activity is crucial for RAS/MEK-induced colon cancer proliferation (74, 75).

### PI3K-AKT-mTOR Pathway

The PI3K-AKT-mTOR signaling pathway is another crucial non-canonical activator of GLI1, particularly evident in pancreatic cancers (76). Activation of PI3K-AKT signaling has been found to enhance GLI1 protein stability (77) since AKT is able to extend GLI proteins half-life in the cells by alleviating the inhibitory effect of PKA and facilitates nuclear translocation. Another mechanism of PI3K signaling activating GLI1 is *via* members of the ribosomal S6 kinase family (S6K/p70-S6K), which are the downstream effectors of the PI3K-AKT-mTOR axis. Activated S6K1 promotes GLI1 disassociation from SUFU by phosphorylating GLI1 at Serine residue at position 84, increasing GLI1 transcriptional activity (78). Additionally, p70-S6K2 has been shown to inhibit GSK3 by phosphorylating GLI1at Ser9, leading to decreases of GSK3b-mediated GLI1 degradation (79).

### TGFβ Pathway

TGFβ is a multifunctioning cytokine that has been implicated in nearly all the key steps of tumorigenesis, tumor maintenance and advanced metastasis (80). In brief, TGFβ is secreted as a latent complex and sequestered in the extracellular matrix until activated. Once biologically available to its target cells, TGFβ binds its type 2 receptor (TGFBR2), leading to the recruitment of its type 1 receptor (TGFBR1) and subsequent downstream signaling resulting in nuclear localization of the SMAD2/3/4 complex (81). In advanced pancreatic cancer, TGFβ signaling contributes to a metastatic phenotype (82). GLI1 as an effector of TGFβ signaling as it interacts with SMAD proteins to induce a subset of TGFβ-inducible target genes, including BCL2, IL7, and Cyclin D1 (83). In the mouse model of PDAC, SMO-independent GLI1 activation promotes transformation and requires both TGFβ and KRAS signaling (84) where inhibition of TGFβ by TbRI antagonist SD208 significantly reduces tumor burden and increases infiltration of lymphocytes.

### Other Pathways

C-MYC, which is frequently over-amplified in colorectal cancer, has been confirmed to be another oncogene that activates GLI1 independently from Hh ligand-mediated signaling (85). C-MYC is a transcriptional activator of GLI1. C-MYC-GLI1 activated pathway could be blocked by small molecule inhibitors targeting either protein, downregulating GLI1 expression and, in turn, inducing cell apoptosis of colorectal cells. Similarly, aberrant expression of oncogenic EGFR, which is responsible for the over-activation of GLI1 through RAS-RAF-MEK pathway, promotes colorectal cancer metastasis and chemotherapeutic resistance. In triple negative breast cancer, elevated expression of GLI1 is driven by VEGF/NRP2 and α6β1 pathway results in an autocrine feedback loop with GLI1 enhancing the expression of NRP2 (86). Atypical protein kinase C iota/lambda (aPKC) has been identified as a novel regulator of GLI, and like the VEGF/NRP2 pathway, results in a positive feedback loop enhancing GLI1 overexpression in basal cell carcinoma (87) and has been also observed in drosophila (88). An interesting connection between GLI1 and p53 has also been reported because of loss of p53 results in aberrant GLI1 expression (89). Genetic mutations of aforementioned pathway genes have been shown to drive GLI1 expression in multiple types of cancer and cancer precursor diseases (90–95). An interesting GLI1 genetic translocation was first noted in 2004 when five pericytomas had an ACTB-GLI fusion transcript t(7;12) (96), with an additional three patients reported on 15 years later (97). Additional fusions were later observed with ACTB1/MALAT1/PTCH1-GLI1, which were associated with metastasis to the lung/lymph node in three of the patients (98).

## GLI1 in Cancer

While GLI1 and GLI2 are both transcriptional activators, GLI1 can be thought of as the primary effector of Hh signaling since GLI1 is a transcriptional target of GLI2, which may amplify Hh-induced, GLI2-mediated transcription of GLI1 target genes (99–102). As previously stated, GLI1 induced by Hh signaling is important in the regulation of cellular proliferation, stemness, cell fate determination, and cellular survival in a variety of organs (36, 103); however, its aberrant activation has been associated with many human cancers (104). For example, GLI1 is amplified in glioma (37), osteosarcoma, and rhabdomyosarcoma (105). Mutations in PTCH or SMO are also prevalent in basal cell carcinomas, medulloblastomas, and cancers of the esophagus and bladder (102), and sustained and activated Hh-Gli signaling has led to the development of medulloblastomas in PTCH heterozygous mice (106). Melanomas and carcinomas of the prostate have further demonstrated a need for elevated Hh-Gli signaling, since inhibition by cyclopamine (a SMO inhibitor) can result in reduction for these types of cancers (107, 108).

Although GLI1 plays a key role in canonically activated Hh cancers (103, 109), non-canonical oncogenic activation (CMYC, RAS/RAF, TGFβ, etc) is critical to address as well (110). For example, in gastrointestinal (GI) cancers, over-activation of GLI1 is driven by KRAS/BRAF mutation (102). It has recently been suggested that oncogenic GLI1 progresses during colon carcinogenesis (111, 112) and in metastatic disease (31), whereas in normal colonic tissue, Hh-GLI is strictly involved in differentiation (59, 113).

### Cancer Stem Cells and Colorectal Cancer

Colorectal cancer (CRC) is still one of the most common gastrointestinal cancers worldwide and results in approximately 33% mortality rate, despite several therapeutic advancements (114). The most important prognostic indicator is stage at diagnosis. The 5-year relative survival of patients diagnosed with CRC is 90% for patients with localized disease (non-metastatic), whereas clinical statistics shows less than 5% 5-year survival for metastatic CRC (115, 116). Therefore, oncogenic drivers of metastasis promote a significant problem to both CRC patients and clinicians (63, 117). The mechanism for CRC progression toward metastasis is multifactorial, with age, dietary habits, genetic alteration (mutational activation of oncogenes and inhibition of several tumor suppressor genes), intensity of epithelial-to-mesenchymal transformation (EMT), angiogenesis in tumor growth, and response to the therapeutic treatment all playing roles in the progression of disease (118, 119). Various gene mutations (KRAS, MYB, and BRAF) and gene abnormal amplification (CMYC and EGFR) have been associated with the molecular mechanisms underlying the development of CRC, all of which can result in non-canonical activation of GLI1 (120, 121). Another complication for studying and treating CRC is the heterogeneity of the disease. This heterogeneity is driven by the by pluripotent, self-renewing cancer stem cells (CSCs) which have unlimited self-renewal through symmetric cell division, and have the ability to give rise to progeny cells through asymmetric division, and an innate resistance to cytotoxic therapeutics (122). Additionally, may publications have implicated Wnt, Notch, Hh, and/or TGFβ signaling pathways in proliferation and maintenance of CSCs, and dysregulation of these pathways might cause the development of CRC (123–127). All of these pathways drive GLI1 expression, defining GLI1 as a cancer stem cell marker in multiple types of cancer, including colorectal (128–131).

### Metastasis and Pancreatic Cancer

Pancreatic ductal adenocarcinoma (PDAC) is one of the deadliest types of cancer in the United States, with a 5-year survival rate of less than 3.5% (132, 133). Removal of the tumor is the only potentially curative treatment to date, but this is not achievable for over 85% patients due to non-resectable cases like early-stage metastasis or complicated primary site (80). KRAS over-activation mutations play a major role in initiating the transformation from precursor lesions termed “pancreatic intraepithelial neo-plasias” (PanINs) to PDAC and promote cancer development and metastasis (134–136). In pancreatic cancer, KRAS mutations are present in 90% of cases (137). Multiple mutation types exist, with codon-13 (G13D) or -61 (Q61L or Q61H) occurring less frequently and 95% of KRAS mutations occurring at codon-12. Single-nucleotide mutations on codon-12 result in eight different amino acid substitutions, with G12D the predominant mutation (51%), G12V (30%), G12R (12%), G12C (2%), G12S (2%), G12A (2%), G12L/F (1%) (11, 14). These missense mutations enhance the level of GTP-bound active KRAS due to impairing intrinsic and GTPase-activating protein-mediated GTP hydrolysis, resulting in over-activating downstream signaling, increasing cell growth and survival, leading to neoplastic transformation (138–140). For patients with locally advanced and/or metastatic PDAC, a G12D KRAS mutation within the primary tumor is an independent prognostic factor that results in significantly decreased overall survival, including those within the subgroup that receive chemotherapy (141). Pancreatic cancer with activating mutations in KRAS or BRAF occur frequently, and oncogenic pathways like RAS/RAF/MEK/ERK, the PI3K-AKT-mTOR, and TGFβ signaling converge on the activation of GLI1, promoting cellular proliferation, tumor progression, chemotherapeutic resistance, and early metastasis (142, 143).

### Radiosensitivity, Heterogeneity, and Brain Cancer

Glioblastoma multiforme (GBM) is the most aggressive and most common type of brain tumor. The standard of care for patients with GBM is maximum safe surgical resection followed by concurrent temozolamide (TMZ) and radiation therapy (144). TMZ is an alkylating agent that results in the transport of methyl groups to guanine and adenine, resulting in DNA damage and eventual cell cycle arrest and apoptosis. TMZ also acts as a radiation-sensitizer to enhance the DNA damage induced by the ionizing radiation. Individuals receiving this standard treatment have a median survival time between 12 and 15 months and have an average 5-year survival of 5% in the United States. Unfortunately, approximately 50% of patients do not respond to the standard of care regimen (145). Most of these cases are the result of overexpression of O6-methylguanine-DNA methyl-transferase (MGMT), a protein that directly counters the methyl damage caused by TMZ (146). In fact, hypomethylation of the MGMT promoter is a biomarker for aggressiveness of disease and poor response to therapy (147). GLI1 was recently identified as positive regulator of MGMT, having several putative binding sites in the MGMT promoter region (148). Aberrant activation of GLI family members has been linked to chemotherapeutic resistance to TMZ (69). Data set analysis from the Chinese Glioma Genome Atlas (CGGA) indicates that individuals with lower expression of GLI1 (149) have a statistically greater median survival when compared to GLI1 high-expressing patients. Several studies have examined the effect of GLI inhibition in GBM cells *in vitro* and found that treatment with GLI inhibitors, like GANT61, results in decreased expression of MGMT and re-sensitization to TMZ (148, 150, 151).

Neuroblastoma accounts for roughly 8% of all childhood malignancies and up to 15% of all pediatric cancer deaths (152). It is a heterogeneous solid tumor, and the heterogeneity is partially driven by the generation of extrachromosomal circular DNA (eccDNA) (153). eccDNA formation has been linked to the dysregulation of the double-stranded break (DSB) repair mechanism, specifically that which drives non-homologous end-joining (NHEJ) and is produced through R-loop defects or circularization of gene fragments (154). Oncogenic GLI1 drives R-loop formation, and treatment with GANT61 has been shown to decrease the generation of R-loop formation (155), likely additionally reducing the generation of eccDNAs.

## Impact of GLI1 on Biological Processes

### Metastasis and Epithelial-Mesenchymal Transition

EMT is considered to be an important feature in cancer development. This process allows the epithelial cells to undergo various biological changes, transforming them to a mesenchymal cell phenotype characterized by enhanced migration, invasiveness, and resistance to apoptosis. EMT markers, such as snail family of zinc-finger transcription factor 1 (SNAIL1), vimentin, and E-cadherin, are three of the primary factors that regulate the EMT transition. GLI1 can initiate cancer cell EMT by increasing expression of SNAIL1 and vimentin but decreasing E-cadherin, causing β-catenin to migrate into the nucleus and act as a transcription factor, inducing cell transformation (156, 157). Since β-catenin is an important member of the WNT signaling pathway, this results in cross-talk between WNT pathway and Hh pathway, resulting in GLI1 activation (158). Overexpression of GLI1 in colorectal cancer cells induces more invasive growth in organoid 3D cultures as well as in soft agar colony formation (159).

### DNA Damage Repair Response

GLI1 activation has been linked to the DNA damage response (DDR) and promotes chemotherapeutic resistance. Recent studies have demonstrated that loss of either non-homologous end joining(NHEJ) gene DNA Ligase IV (Lig4), or genes involved in homologous recombination (HR) like X-ray cross complementation 2 (XRCC2), and breast cancer growth suppressor protein 2 (BRCA2), or (Lig4/XRCC2) in combination with p53 deficiency results in PTCH-1 downregulation and GLI1 activation (69, 160). DNA damaging agents, such as doxorubicin and cisplatin, induced concomitant expression of p53 and downregulation of GLI1 and its target genes (161). In response to damage, p53-induced cell cycle checkpoints prevents proliferation of damaged cells and provides sufficient time for repair, which is the opposite response that GLI1 promotes (69).

Specific inhibition of GLI1 induces extensive cell death while the inhibition of Hh signaling at the level of SMO did not in colorectal cancers (161). In HT29 cells, inhibition of GLI1 by siRNAs or GANT61 (a small molecule inhibitor) showed increased DNA damage and cell cycle arrest at G1–S and in early S-phase, resultant of down-regulation of cell cycle genes, such as E2F2, cyclin E2, Cdc25a, Cdk2 and cyclin A2, Cdc25c, cyclinB2, Cdc20, Cdc2. Inhibition of GLI1 induces serious DNA damage because it pauses DNA synthesis by impairing the ensemble of DNA licensing pre-complex and accumulates conflicts by head-to-head jam made by DNA and RNA synthesis machinery due to cell cycle arrest (155). Additionally, inhibition of GLI1 not only promotes cell cycle arrest it also impairs cell innate DNA damage response procedure. The DDR machinery is comprised of multiple sensors and repair enzymes that are deployed at various stages of the cell cycle to ensure the maintenance of chromosomal integrity and replicative fidelity. Numerous reports of overexpression of critical DDR component proteins in oncogenic environments indicate that chemo-resistance can arise due to over-activation of the MRE11, Rad50, NBS1 (MRN) complex. A critical component of the MRN complex is the Nijmegen breakage syndrome-1 (NBS1; p95, nibrin) protein, produced by NBS gene. Complexing with MRE11 and RAD50, NBS1 is the first factor to detect and bind to histone H2AX at the site of a DNA lesion which subsequently forms the multimeric MRN complex, initiating the process of DSBs repair (162–164). Overexpression of individual components of the MRN complex has been significantly associated with adverse clinical outcomes due to chemotherapeutic resistance. Therefore, induced novel therapeutic avenue would be to inhibit the DDR mechanism, allowing chemotherapeutic mechanisms that target DNA damage to work more effectively. The challenge, however, is to specifically eliminate DDR in cancer cells without affecting the normal and necessary functions of DDR in non-cancerous cells.

Ataxia-telangiectasia mutated (ATM) is a kinase that regulates a number of substrates, including the phosphorylation of NBS1, which is required to initiate and enhance NBS1’s DDR activity. As such, several programs have attempted to develop various ATM inhibitors aimed to inhibit DDR (165). Unfortunately, ATM itself is not a specific therapeutic target because of its multiple domained nature, critical kinase function in normal cellular processes, and essential role in the maintenance of chromosome integrity at all phases of the cell cycle (166). Some studies reported that the level of phosphorylated NBS1 (Ser343), which is regulated by its upstream kinase ATM/ATR, is a critical phosphorylation status thought to increases DNA damage response and promotes cell survival. To test this theory, our lab overexpressed wild type NBS1, domain-negative NBS1 (S343A), or phospho-mimic NBS1 (S343E) in HT29 cells. Overexpression of any NBS1 vector rescued ~25% of cells from apoptosis mediated by GLI inhibition. Surprisingly, the overexpression of S343E, S343A, or total NBS1 was not statistically different from one another, indicating that total levels of NBS1, elevated by GLI1 transcription, rather than the phosphorylation status, were responsible for protection from GLI inhibition-induced apoptosis (64). Since GLI1 is not typically expressed by differentiated cells, targeting oncogenic expression of GLI1 would result in fewer off-target effects and provide a specific therapeutic strategy.

## GLI1 Inhibitors

Most of the efforts to-date have typically focused on targeting GLI inhibition through the canonical Hh pathway, targeting upstream regulators like SMO, and subsequently sequestering GLI1 in the cytoplasm. Five SMO inhibitors have been approved by the FDA for clinical trials: vismodegib (GDC-0449), sonidegib (NPV-LDE-225), saridegib (IPI-926), BMS-833923, glasdegib (PF-04449913), and taladegib (LY2940680) (167). Variable success using SMO inhibitors has been demonstrated across a variety of different cancer types in preclinical models (30, 31, 107, 168–171) and clinical models (172–177). This is due to the predominant dependence of certain types of human cancers on canonical Hh signaling, such as basal cell carcinoma (173, 177), and medulloblastoma (172). However, clinical trials in most solid tumors have failed, likely because of aforementioned non-canonical activation pathways (i.e., RAS-ERK, PI3K-AKT-mTORS6K1 signaling, p53 loss, epigenetic alterations, etc.). Therefore, direct targeting of GLI might represent a better choice to improve the antitumor activity of these drugs in such cases.

The library of GLI1 antagonists is not as extensive as that for SMO. The most commonly used small molecules are GANT58 and GANT61, which were identified in a cell-based GLI-dependent luciferase screening system (178). These two compounds belong to different chemical classes, with GANT61 being a hexahydropyrimidine derivative and GANT58 possessing a thiophene core with four pyridine rings. Compared to GANT58, GANT61 is more specific toward GLI proteins and effectively reduces GLI1 and GLI2 DNA-binding ability, inhibiting the Hh pathway with a half maximal effective concentration (EC50) of 5 μM in GLI1-expressing HEK293T cells (26). GANT61 binds to the GLI1 protein between ZF2 and ZF3, by interacting with Glu119 and Glu167, as demonstrated by *in silico* docking on the crystal structure of the ZF domain of GLI1 bound to DNA (119). Experimental analysis shows that mutation of the predicted binding sites significantly reduces GANT61-GLI binding affinity. The GANT61 binding site is different from the GLI DNA-binding region, and the inhibitor is not able to bind to other ZF transcription factors such as KLF4 or TFIIβ (26, 119). Unfortunately, GANT61 is not usable as a translational therapeutic as it is unstable and has poor PK properties (179).

Using GANT61 as an initial scaffold, Southern Research has recently developed a novel GLI1 inhibitor (SRI-38832) that has better PK properties and has shown efficacy *in vivo* (64). Additionally, there are several promising compounds showing the biological activity of GLI inhibition (180), arsenic trioxide (ATO), originally approved by the FDA for the treatment of acute promyelocytic leulemia, has been shown to inhibit GLI proteins by binding to GLI proteins and enhancing degradation (181). ATO is currently being tested in multiple clinical trials ranging from phase I to phase IV for either solid tumors and hematologic malignancies. However, recent reports indicate lack of efficacy against small cell lung cancer (182). Polyunsaturated fatty acids (PUFAs) have also been reported to repress GLI1 expression by stimulating GLI1 suppressor, nuclear factor of activated T cells 1 (NFATc1) expression (183). Glabrescione B (GlaB), an isoflavone naturally found in the seeds of Derris glabrescens, is able to bind the GLI1 ZF domain, thereby diminishing GLI1/DNA interaction (184). Leadiant Biosciences used Glabrescione B as their scaffold for generating a pool of compounds for GLI1 inhibition (185, 186). Computational modeling of the DNA/GLI1 protein interaction has also been used to develop an 8-hydroxyquinolines as a GLI1 inhibitors, with similar scaffolds as Lediant Bioscience’s compounds (187). Finally, the Hedgehog pathway inhibitors (HPIs) including HPI-1, HPI-2, HPI-3, and HPI-4, were identified with a high-throughput screening for compounds capable of abolishing the Hh target gene expression induced by the SMO agonist SAG (188). HPI-1 can suppress Hh pathway activation, likely through targeting a posttranslational modification of the GLI proteins and/or an interaction between the transcription factor and a co-factor (189). The detailed mechanisms of action have not yet been completely unraveled.

One specific problem often encountered is the non-specificity of developed compounds claiming to be specific for GLI1 (i.e., also inhibit GLI2 and decrease GLI2 protein/messenger expression). The homology of GLI1 and GLI2, along with the similarities in the promotor recognition sequence makes it difficult to design an inhibitor of one without inadvertently targeting the other. Computational modeling and structural biology (NMR; crystallography) can help to resolve the challenge of non-specificity.

## Conclusions

GLI1 exists at the conjunction of multiple oncogenic pathways outside of the canonically understood hedgehog pathway. In the scope of oncogenesis, GLI1 activation is particularly dominant in subsets of a number of cancer types because parallel non-canonical pathways outside of hedgehog signaling influence GLI1 function. Additionally, the list of GLI1 transcriptional targets continues to expand, encompassing cell cycle regulators (Cdt1), DNA damage repair proteins (NBS1), and proliferation (FOXM1). In certain cancers, it promotes a dedifferentiation to a more stem-like phenotype. Because of GLI1’s regulatory fluidity, targeting upstream pathway members is often an exercise in futility, as seen by the failure of SMO inhibitors, for example. For this reason, GLI1 is a significant therapeutic target for the treatment of multiple cancer types.

Whether overexpressed due to canonical, non-canonical, or genetic mutation, elevated GLI1 expression drives several of the hallmarks of cancer including DNA damage repair, cell proliferation, and metastasis. Rather than target upstream regulators of GLI, targeting the distal effector provides the greatest potential for therapeutic benefit. Since GLI1 is canonically active in embryonic development, with minimal basal expression in differentiated cells, it 1) serves as a biomarker for de-differentiation in cancer cells, particularly those refractory to treatment and 2) provides a prominent target not readily expressed in most non-cancerous tissue. As such, by targeting the downstream effector (GLI1) rather than upstream activators, we can effectively inhibit the oncogenesis driven by aberrant GLI1 activation, and promote cancer-specific DNA damage. While many promising drug discovery campaigns are developing and looking for novel GLI1 inhibitors, more work needs to be done to develop a potent, specific inhibitory compound.

## Author Contributions

JA and RZ compiled the information and wrote the manuscript. RB reviewed and corrected the manuscript. JA and RZ have contributed equally to this work and share first authorship. All authors contributed to the article and approved the submitted version.

## Funding

This work was supported by The National Institutes of Health [5RO1CA183921-05].

## Conflict of Interest

The authors declare that the research was conducted in the absence of any commercial or financial relationships that could be construed as a potential conflict of interest.
